# Influence of Change in Echo Intensity of the Pretalar Fat Pad in Young Individuals With Chronic Ankle Instability

**DOI:** 10.7759/cureus.73114

**Published:** 2024-11-06

**Authors:** Kakeru Hasegawa, Masahiko Wakasa, Kazuki Okura, Akira Saito, Minoru Kimoto, Yoshino Terui

**Affiliations:** 1 Department of Physical Therapy, Akita University Graduate School of Health Sciences, Akita, JPN; 2 Department of Rehabilitation, Akita University Hospital, Akita, JPN

**Keywords:** ankle sprains, b-mode ultrasonography, chronic ankle instability, echo intensity, pretalar fat pad

## Abstract

This study aimed to investigate the echo intensity (EI) of the pretalar fat pad (PFP) in young individuals with chronic ankle instability (CAI) and clarify the relationship between changes in the PFP and the clinical characteristics of CAI. Using the Identification of Functional Ankle Instability (IdFAI) scores, 26 limbs of 15 participants were divided into CAI (IdFAI score ≥11, male: eight limbs, female: six limbs, age: 21±1 years) and normal (IdFAI score <11, male: nine limbs, female: three limbs, age: 20±1 years) groups. The EI of the PFP was measured, and the luminosity ratio (LR) to the EI of the subcutaneous adipose tissue was calculated. To evaluate ankle joint stability and function, the fibulo-talar separation rate and weight-bearing lunge test (WBLT) values were measured. These parameters were compared between the two groups, and their correlations were statistically analyzed. The median IdFAI score in the CAI group was 15.5. The LR of the PFP was significantly higher in the CAI group than in the normal group (P<0.01). The fibulo-talar separation rate values were significantly higher (P=0.006), and the WBLT values were lower (P=0.011) in the CAI group. A moderate negative correlation was observed between the LR and WBLT values (*r* =−0.44, *P*=0.03). The LR of the PFP was high in the CAI group and was related to limited dorsiflexion of the ankle joint in the loaded position. Evaluating the entire ankle joint, including the PFP, is important, even for young individuals with mild CAI.

## Introduction

Ankle sprains are a high-incidence trauma [1]. More than 75% of these injuries are due to forced inversion, and approximately 73% are associated with a rupture of the anterior talofibular ligament [2]. Additionally, the recurrence rate is reportedly as high as 56−74%, and it is known that chronic ankle instability (CAI) is likely to occur after repeated ankle sprains [3,4]. A common symptom of CAI is limited dorsiflexion range of motion of the ankle joint [5]. Impingement by soft tissues or osteophytes in the anterior ankle region is considered a contributing factor to CAI [6].

The anterior portion of the ankle joint contains a pretalar fat pad (PFP), synovium, and collagenous tissue [7]. The PFP is pinched by the ankle joint at a 15° dorsiflexion angle and is considered to be a cause of anterior ankle impingement [8]. However, changes in the PFP in individuals with CAI and its relationship with clinical symptoms have not been studied. Therefore, evaluating the PFP in individuals with CAI may be necessary to clarify the pathogenesis associated with the limited range of motion.

Recently, ultrasonography has been widely used to evaluate both morphology and echo intensity (EI). EI is reportedly valuable for the noninvasive evaluation of skeletal muscles and adipose tissues and may even reveal histological changes [9,10]. This study aimed to compare the EI of the PFP between individuals with CAI and healthy controls and clarify the relationship between EI changes in the PFP and clinical symptoms associated with CAI.

## Materials and methods

**Figure 1:**
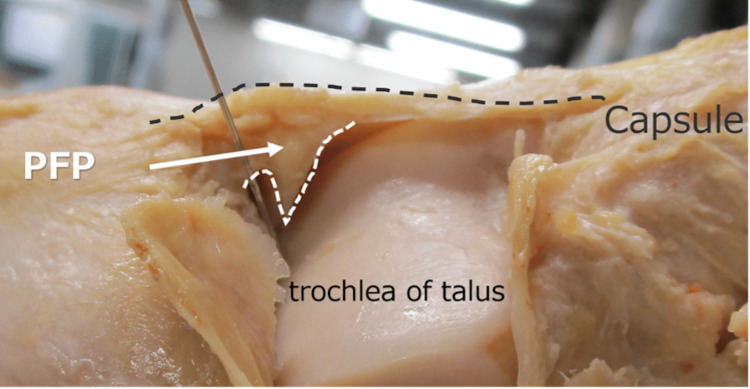
Anatomical location of the pretalar fat pad (PFP). The PFP is located anterior to the talus and within the joint capsule. (Photographed by the author for this study)

Fifteen young adults (eight males, seven females; mean age, 21.0±1.0 years; mean body mass index (BMI), 21.0±1.5 kg/m²) recruited using community-based advertisements were enrolled in this study. Among the 30 limbs of the 15 participants, four limbs that experienced trauma within a month were excluded. Consequently, 26 limbs (right, 13 limbs; left, 13 limbs; male, 17 limbs; female, nine limbs) were included in this study. The ankle status of all participants (limbs) was evaluated using the Identification of the Function of Ankle Instability (IdFAI) [11] questionnaire recommended by the International Ankle Consortium, following Gurav’s criteria [12]. A diagnosis of CAI was made according to the following criteria: a score of ≥11 of 30 points was considered a CAI limb, and a score of ≤10 was considered a healthy limb (Figure 1) [11].

Following the Declaration of Helsinki, the purpose and methods of this study were explained to the participants in advance using the prescribed form, and their consent to participate was obtained in writing. This study was conducted at Akita University Hospital (Akita, Japan) and approved by the Ethics Committee of the Akita University Graduate School of Medicine (approval no. 2743).

The participants’ ages, heights, weights, and BMIs were collected from the questionnaire. The weight-bearing lunge test (WBLT), which evaluates ankle dorsiflexion function under a loading status, was performed as follows: The participant was instructed to stand in front of a wall and step forward with the measuring limb until the anterior knee parts were attached to the wall. The line between the center of the heel and the big toe of the measuring limb was maintained perpendicular to the wall. Then, the participant was instructed to lean the lower leg maximally forward while maintaining anterior knee contact on the wall with the heel on the floor. The distance between the tip of the big toe and the wall was measured with tape in 0.5 cm increments as the value of the WBLT. Measurements were performed twice on the same day by the same examiner, and the average value was obtained.

The fibulo-talar separation rate (FTR) was measured as a parameter for assessing ankle instability. The heel and thigh were placed on a table with the knee extended. The anterior talofibular ligament was observed using an ultrasound device (HI VISION Avius; Hitachi, Ltd., Tokyo, Japan) and a linear-type probe (EUP-L65; Hitachi, Ltd.). The distance between the external talipes and fibula (Distance #1) was measured as the length of the ligament. In cases of an obscure ligament, the distance between the typical bony landmarks was measured as the length. Then, a force perpendicular to the floor was manually applied at a position 7 cm proximal to the medial malleolus and the distance between the talus and fibula under manual stress (Distance #2). The FTR was measured using the following formula: ((Distance #2 - Distance #1) / Distance #1) × 100 (%). Measurements were taken thrice by the same examiner on the same day, and the average value was used.

The entire image and its EI were observed using an ultrasound imaging system (HI VISION Avius; Hitachi, Ltd., Tokyo, Japan) with a 10 MHz linear type probe (EUP-L65; Hitachi, Ltd., Tokyo, Japan). The gain was set to 20, the dynamic range was set at 65, and the focus was set to 2.0 cm. The PFP was observed under the maximum plantarflexed position of the ankle, enabling observation of the entire PFP, and the probe was placed in front of the ankle joint parallel to the long axis of the lower leg. The EI of the PFP was measured using the image editing program ImageJ (National Institutes of Health) with 8-bit grayscale, according to the method by Wu et al. [13]. The ultrasound luminosity ratio (LR) was calculated using the following formula: LR=EI of PFP / EI of the subcutaneous adipose tissue [13].

EZR version 4.0.3 (Saitama Medical Center, Jichi Medical University, Saitama, Japan) was used for all statistical analyses. The normality of age, BMI, IdFAI score, WBLT, FTR, and LR was examined using the Shapiro-Wilk test. For comparisons between the two groups, an unpaired t-test was used when normality was present, and the Mann-Whitney U-test was used when normality was absent. The chi-square test was used to compare the sex and frequency of PFP morphological abnormalities between the two groups. Spearman’s rank correlation coefficient was used to examine the correlation between LR and the WBLT. Correlation coefficients of r=0.7−1.0 were considered highly correlated, r=0.4−0.7 were moderately correlated, r=0.2−0.4 were weakly correlated, and r < 0.2 signified almost no correlation. The significance level was set at 5%. Intraclass correlation coefficients (1, 3) were calculated for each measure.

## Results

The characteristics of the CAI and control groups are shown in Table 1. Of the 26 limbs, 14 were classified into the CAI group, and 12 were classified into the healthy group. The IdFAI scores were significantly higher in the CAI group than in the healthy group. Age, sex distribution, and BMI were not significantly different between the two groups.

**Table 1:** Characteristics of the chronic ankle instability (CAI) and healthy groups *: mean ± standard deviation, **: median ± interquartile range; BMI: body mass index; IdFAI: Identification of Functional Ankle Instability

	CAI group (n=14)	Normal group (n=12)	P-value
Age (years)*	21.4 ± 1.5	21.4 ± 1.6	0.56
Male: Female *	8:6	9:3	0.08
BMI (kg/m²) *	21.0 ± 1.5	20.8 ± 1.6	0.65
IdFAI (point) **	15.5 (14.2, 17.0)	2.0 (1.0, 3.0)	< 0.001

**Figure 2:**
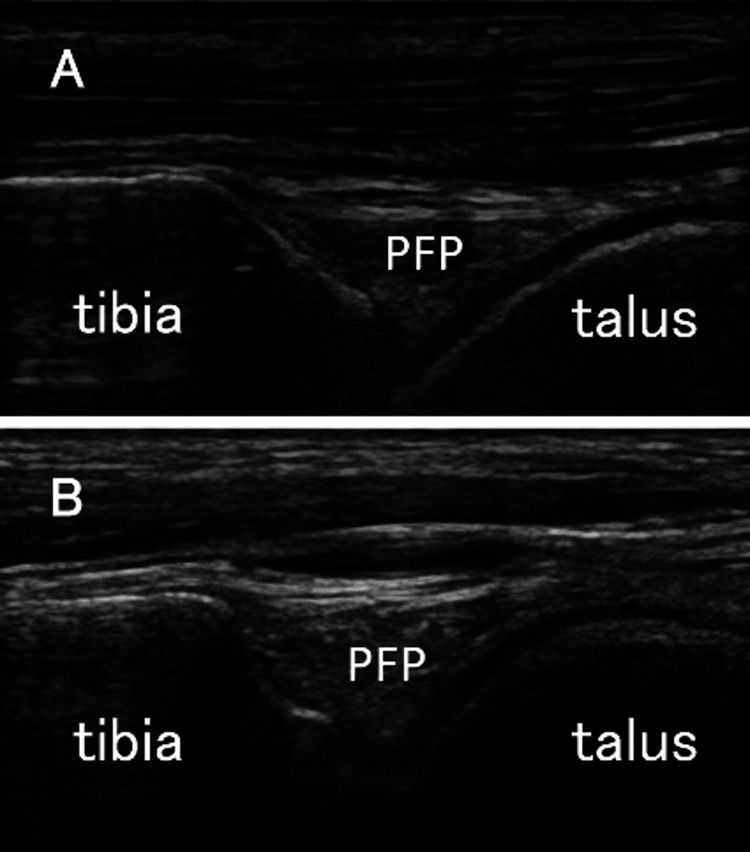
Ultrasound images of the pretalar fat pad (PFP). Ultrasound images of the pretalar fat pad (PFP) in the healthy group (A) and chronic ankle instability (CAI) group (B). The CAI group has higher ultrasound intensity and more indistinct contours than the healthy group.

**Figure 3:**
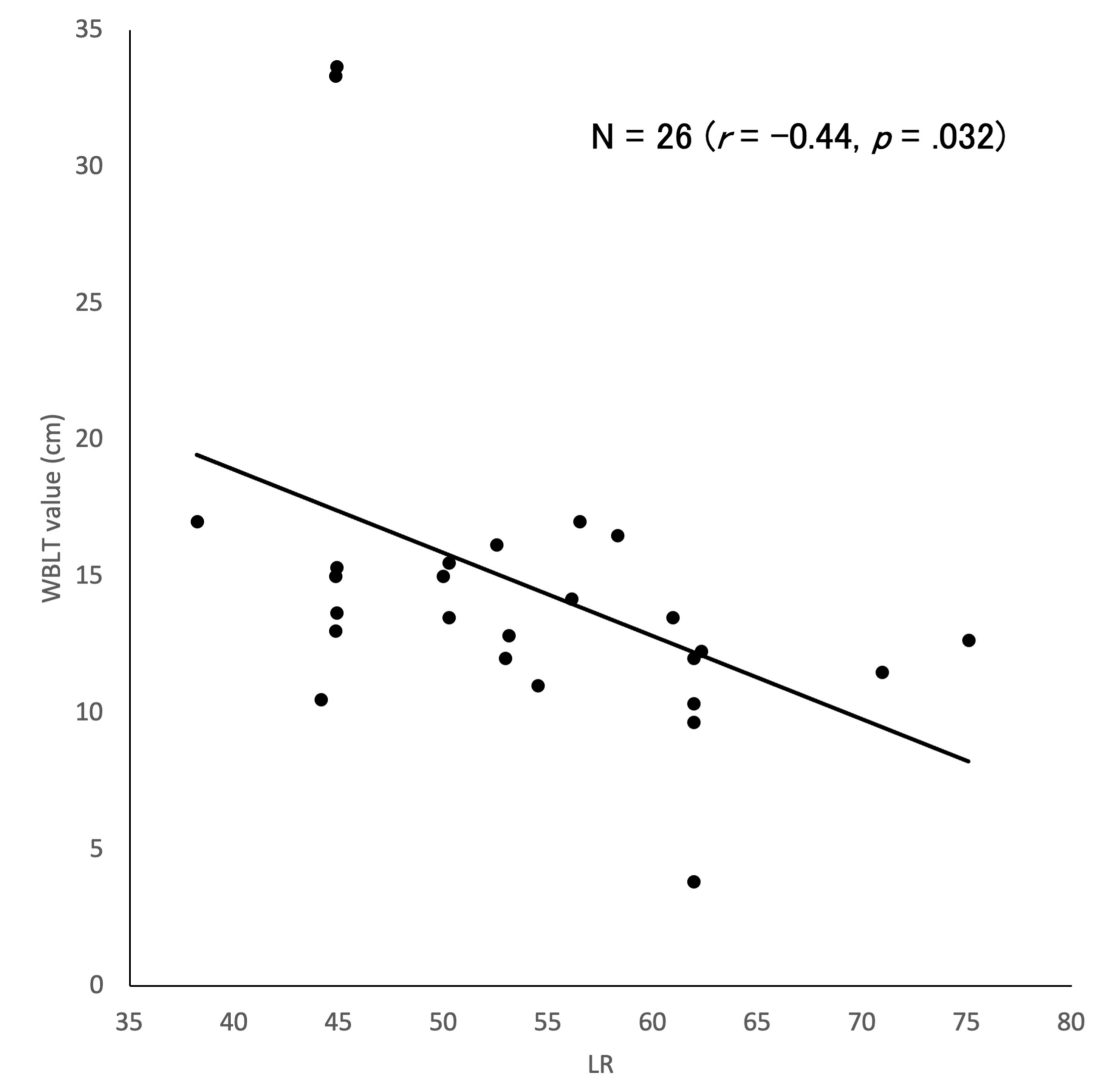
Correlation between weight-bearing lunge test values and ultrasound radiance of the pretalar fat pad (corrected for subcutaneous fat values) in chronic ankle instability. WBLT: weight-bearing lunge test; LR: luminosity ratio

The WBLT value was significantly lower (P=0.011, Table 2), and the FTR value was significantly higher in the CAI group than in the healthy group (P=0.006, Table 2). As shown in Figure 2, ultrasonic observation revealed an obscure margin of the PFP in three of the 14 limbs (21%) in the CAI group. No morphological changes were observed in the control group. The LR was significantly higher in the CAI group than in the healthy group (Table 2). The frequency of morphological changes did not differ between the two groups. However, there was a moderate negative correlation between LR and WBLT (r=−0.44, P=0.03, Figure 3). The intraclass correlation coefficients (1, 3) for LR, WBLT, and FTR were 0.92, 0.90, and 0.89, respectively, indicating high reliability for each measure.

**Table 2:** Results of WBLT, talofibular distance opening ratio, morphological abnormalities of PFP, and luminosity ratio. *: mean ± standard deviation; **: median ± interquartile range; WBLT: weight-bearing lunge test; FTR: fibulo-talar separation rate; LR: luminosity ratio

	Chronic ankle instability group (n=14)	Normal group (n=12)	P-value
WBLT (cm) *	11.9 ± 2.7	17.9 ± 7.0	0.011
FTR (%) **.	12.2 (10.1, 15.7)	5.0 (3.6, 6.6)	0.006
Morphological abnormality (n) *	3	0	0.22
LR *	22.8 ± 0.8	24.3 ± 1.4	0.007

## Discussion

This study compared the EI of the PFP between individuals with CAI and healthy controls and assessed the relationship between EI changes in the PFP and clinical symptoms associated with CAI. The IdFAI scores were significantly higher in the CAI group than in the healthy group. The LR of the PFP was significantly higher in the CAI group than in the normal group, and a moderate negative correlation was observed between the LR and WBLT values. This study is the first to demonstrate an association between the EI values of the PFP and WBLT, suggesting that changes in the PFP, such as fibrosis, may contribute to dorsiflexion limitations in patients with CAI.

Patients with CAI are reportedly prone to anterior impingement due to soft tissues or osteophytes at the anterior ankle joint [4,14-16]. Odak et al. reported that 63% of patients with CAI had soft tissue impingement around the ankle joint [6]. Anterior impingement by hypertrophic synovium following CAI has also been observed on magnetic resonance imaging [17]. This suggests that recurrent sprains [18] and instability [19] seen in CAI might cause chronic changes in the anterior area of the ankle joint. In this study, the high EI of the PFP associated with CAI was a characteristic finding. Several studies on high ultrasonic EI and its pathogenesis have been reported. Regarding the muscular tissue, high EI has been observed in necrosis or inflammatory conditions [20]. In children with muscular dystrophy, some authors observed high EI in the quadriceps muscles and considered muscle fibrosis as the cause based on pathological findings [9,10]. A study on patients with knee arthritis reported that high EI and decreased body fat morphology were associated with decreased range of motion [21]. Additionally, high EI may resemble the histological changes described in fat bodies and is characterized by inflammation, swelling, hypertrophy, fibrosis, and calcification [22,23]. Similarly, in individuals with CAI, fibrosis may occur in the PFP at the anterior part of the ankle joint, resulting in a high EI.

The FTR, a parameter of ankle instability, was high in the CAI group (approximately 12%). In previous studies, the FTR, or stretched rate, of the anterior talofibular ligament ranged from 11-42% [22,23]. The FTR of individuals with CAI in the present study was relatively low, although it was significantly higher than that of the healthy participants. Furthermore, the median IdFAI score, a subjective index of instability in this study, was also relatively low (50% of the full score). Individuals in the CAI group were considered to have mild-to-moderate CAI. It should be stressed that chronic changes in ankle PFP occur, even in individuals with mild or moderate CAI.

The ankle dorsiflexion range of motion during non-loading is commonly examined in CAI, and various degrees of dorsiflexion limitations have been reported [5,24]. Recently, dynamic measures of ankle function in the loading position, such as the WBLT examined in this study, have replaced conventional methods and have been shown to have good inter- and intra-examiner reliabilities [25]. The WBLT features a significant minimum detectable change of 1.8 cm and is highly sensitive [25]. In the present study, the difference in WBLT values between the two groups was approximately 6.0 cm, which means that clinically meaningful differences were detected in patients with CAI. Furthermore, we found a moderate negative correlation between the EI values of the PFP and WBLT values, indicating that changes such as fibrosis of the PFP in the anterior ankle joint are associated with the dorsiflexion function of the ankle joint.

The limited dorsiflexion range of motion in patients with CAI is thought to be caused by the inhibition of normal bone motion, such as limited posterior sliding of the talus [26,27] and an anterior deviation of the talus [13,18]. Based on these facts, it is presumed that ankle instability occurs first, leading to degenerative changes in the soft tissues, including fibrosis of the PFP, which may affect the motion of the talus. Dynamic observation of the entire ankle joint, including the PFP, could be valuable in understanding and treating patients with CAI.

This study had some limitations. First, we could not examine the histological findings associated with the high EI of the PFP. Therefore, using magnetic resonance imaging as a noninvasive method of investigation may be useful. Second, ultrasonic observation of the PFP was performed in the fully plantarflexed position without any joint motion. Given that the PFP is tissue that is in direct contact with the talus, dynamic observation may be informative. However, dynamic observation of the PFP using ultrasound is technically difficult because of the forward bowstring of the tendons, including the tibialis anterior. Thus, it is necessary to develop a device that can observe PFP during ankle motion.

## Conclusions

We evaluated ankle joint function under WBLT and measured the ultrasound EI of the PFP within the ankle joint in young adults with CAI. Even in young patients with mild or moderate CAI, the EIs of the PFP were high, a limited dorsiflexed motion range was evident, and the findings were correlated. These findings indicate that evaluating the entire ankle joint, including the PFP, is necessary, even in individuals with mild to moderate CAI. Additionally, treatment such as range of motion training targeting the PFP is necessary.
